# New histopathologic and ultrastructural findings in Reis-Bücklers corneal dystrophy caused by the Arg124Leu mutation of *TGFBI* gene

**DOI:** 10.1186/s12886-016-0325-y

**Published:** 2016-09-02

**Authors:** Wen-Ya Qiu, Li-Bin Zheng, Fei Pan, Bei-Bei Wang, Yu-Feng Yao

**Affiliations:** 1 Department of Ophthalmology, Affiliated Sir Run Run Shaw Hospital, Zhejiang University School of Medicine, 3 Qingchun Road East, Hangzhou, 310016 Zhejiang People’s Republic of China; 2 Key laboratory of Biotherapy of Zhejiang Province, Affiliated Sir Run Run Shaw Hospital, Zhejiang University School of Medicine, 3 Qingchun Road East, Hangzhou, 310016 Zhejiang People’s Republic of China; 3 Core Facilities, Zhejiang University School of Medicine, 866 Yuhangtang Road, Hangzhou, 310058 Zhejiang People’s Republic of China

**Keywords:** Reis-Bücklers corneal dystrophy, Corneal Bowman’s layer, Transforming growth factor induced gene, Deep anterior lamellar keratoplasty, Keratocyte

## Abstract

**Background:**

Reis-Bücklers corneal dystrophy (RBCD) was consistently reported as a corneal dystrophy only affected Bowman’s layer and superficial corneal stroma, and superficial keratectomy was a recommendation surgery for treatment in literatures. The study reported new histopathological and ultrastructural findings in RBCD caused by the Arg124Leu mutation of transforming growth factor induced (*TGFBI*) gene in a four-generation Chinese pedigree.

**Methods:**

Subjects including eight patients and seven unaffected family members received slit-lamp biomicroscopy and photography. DNA was obtained from all subjects, and exons 4 and 11 to 14 of *TGFBI* gene were analyzed by polymerase chain reaction and the products were sequenced. Anterior segment optical coherence tomography (AS OCT) and in vivo confocal microscopy were conducted for ten eyes of five patients. Based on the results of AS OCT and in vivo confocal microscopy, deep anterior lamellar keratoplasty (DLKP) using cryopreserved donor cornea was applied for four eyes of four patients. Four lamellar dystrophic corneal buttons were studied by light and transmission electron microscopy, and TGFBI immunohistochemistry.

**Results:**

Eight patients had typical clinical manifestations of RBCD presenting recurrent painful corneal erosion starting in their early first decades, along with age-dependent progressive geographic corneal opacities. *TGFBI* sequencing revealed a heterozygous mutation, Arg124Leu in all eight patients. Anterior segment optical coherence tomography and in vivo confocal microscopy showed the dystrophic deposits involved not only in subepithelial and superficial stroma, but also in mid- or posterior stroma in four examined advanced eyes. Light microscopy showed Bowman’s layer was absent, replaced by abnormal deposits stain bright red with Masson’s trichrome. In superficial cornea, the deposits stacked and produced three to five continuous bands parallel to the corneal collagen lamellae. In mid- to posterior stroma, numerous granular or dot- like aggregates were heavily scattered, and most of them presented around the nuclei of stromal keratocytes. Transmission electron microscopy revealed the multiple electron-dense rod-shaped deposits aggregated and formed a characteristic pattern of three to five continuous bands in superficial cornea, which were similar to those seen under light microscopy. In mid- to posterior stroma, clusters of rod-shaped bodies were scattered extracellular or intracellular of the stromal keratocytes between the stromal lamellae suggesting the close relationship between mutated proteins and keratocyte.

**Conclusions:**

The study offer evidences indicating DLKP is a viable treatment option for advanced RBCD to avoid recurrence, and the mutated TGFBIp in dystrophic corneas are of keratocytes origin.

## Background

Reis-Bücklers corneal dystrophy (RBCD, OMIM 608470) is a rare, bilateral and autosomal dominant inherited disease that primarily affects Bowman’s layer. It was first described by Reis in 1917 as an annular dystrophy [1]. The same German family was re-examined by Bücklers in 1949, and showed that the dystrophy consistently presented geographic opacities seen at the level of Bowman’s layer [1]. Since then, the clinic, histologic, ultrastructural aspects and genetic disorders of RBCD have been described in several papers [2–5]. These reports showed that histopathologic manifestations were stain red, band-shaped granular subepithelial and superficial stromal deposits with Masson trichrome stain. Ultrastructural feature was the presence of “rod -shaped bodies” under transmission electron microscopy. Genetically, RBCD inherited in an autosomal dominant pattern. To date, the Arg124Leu mutation of transforming growth factor induced gene (*TGFBI*, protein: TGFBIp, or keratoepithelin) was the only mutation detected in the serum DNA from patients with RBCD.

TGFBIp, an extracellular matrix protein was first identified from a human lung adenocarcinoma cells that had been treated with TGF-β in 1992 [6]. It mediates integrin binding to extracellular matrix proteins for cell proliferation, adhesion, and migration. In human eye, it was initially found on the external surface of corneal epithelial cells [7], and lately was detected in both endothelium- derived cells and stromal keratocytes [8]. TGFBIp contains four conserved fasciclin 1 (FAS1) domains and one carboxyl-terminal Arg-Gly-Asp (RGD) integrin-binding domain. Mutation of *TGFBI* gene is linked to dystrophic protein deposits in the corneal stroma, the main cause of corneal dystrophy. Specific mutations in two hot spots, Arg-124 in the first FAS1 domain and Arg-555 in the fourth FAS1 domain, are related with several clinical phenotypes including granular, lattice, Avellino, Reis–Bücklers and Thiel–Behnke corneal dystrophy. However, the pathogenesis of TGFBIp-related corneal dystrophy still remains poorly understood, and the origin of dystrophic protein deposits is controversial. Several reports demonstrated the deposits of mutated TGFBIp were of corneal epithelial origin [9–12], whereas a few papers suggested the mutated proteins were secreted by stromal keratocytes [13, 14].

Corneal surgery is required for RBCD eyes for recovery of corneal clarity and visual rehabilitation when the opacity of dystrophic cornea being more dense and severe. Since RBCD has been considered to be a superficial corneal dystrophy, its first recommendation surgical treatment is a minimally invasive procedure such as superficial keratectomy including excimer lasers phototherapeutic keratectomy (PTK) [15–17]. Nevertheless, recurrences can be noted within several months post-operatively. It was reported in a paper, eight (47 %) of 17 eyes with RBCD developed clinically significant recurrence within an average of 21.6 months after PTK [18].

In the present study, we identified a large Han Chinese family containing eight members affected with typical RBCD caused by the R124L mutation of the *TGFBI* Gene. DLKP using cryopreserved donor corneas were applied to treat four dystrophic eyes, and no recurrence was seen in all four eyes during a 4-year period of follow-up. Utilizing these four dystrophic corneal buttons, we did histopathologic studies. The study may offer evidences demonstrating the mutated TGFBIp in RBCD corneas are of keratocyte origin and the disease needs a DLKP for treatment to avoid recurrence.

## Methods

### Subjects

**Fig. 1:**
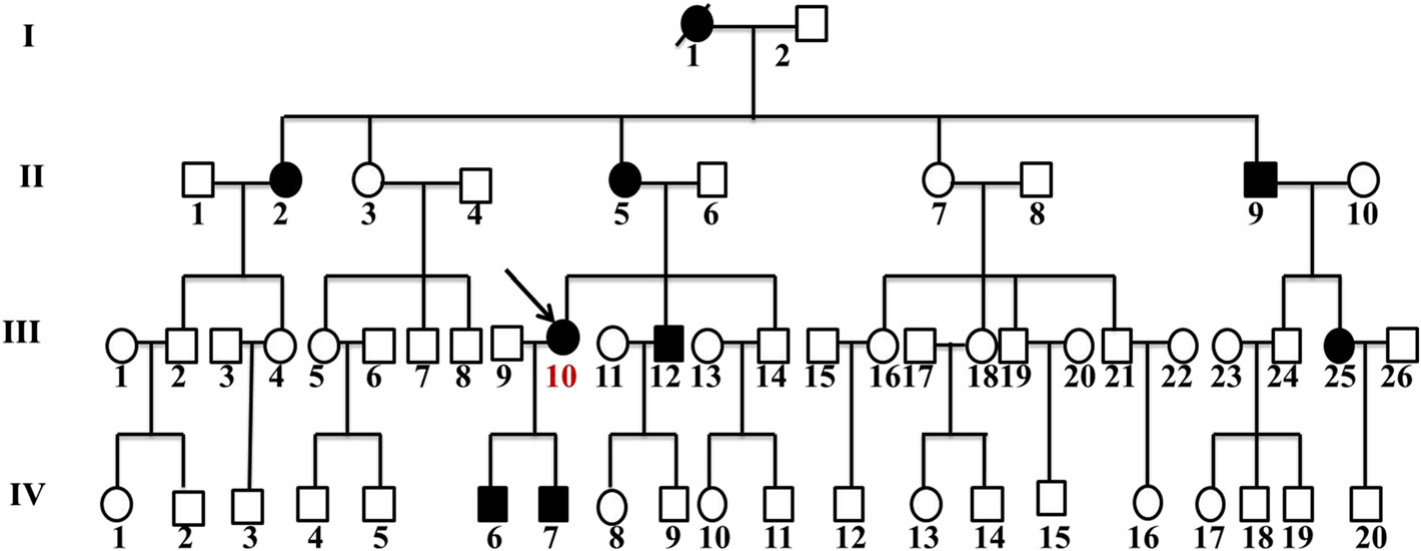
A four-generation pedigree of the studied family affected by Reis-Bücklers corneal dystrophy. Squares and circles indicate males and females respectively. The solid symbols represent affected persons. The arrow denotes the proband. Diagonal line through symbol indicates death

A four-generation Han Chinese family with autosomal dominant inherited corneal dystrophy from Henan province, which is located in eastern central China, was enrolled (Fig. 1). Approved by the institutional ethics committee of Sir Run Run Shaw Hospital, the study included eight corneal dystrophy patients (II:2,II:5,II:9,III:10,III:12,III:25,IV:6,IV:7) and seven unaffected relatives (III:9,III:11,III:14,III:26,IV:8,IV:9,IV:21). Informed consents were obtained from the patients and their family members who participated in this study.

### Clinical examinations

All subjects received detailed clinical examinations including visual acuity and slit lamp biomicroscopy. Examinations were conducted by a same experienced ophthalmologist. Anterior segment optical coherence tomography (AS OCT, Visante™ OCT 1000, Carl Zeiss Meditec AG, Jena, Germany) and in vivo slit scanning confocal microscopy (NIDEK Confoscan 3.0, NIDEK technologies, Vigonza, Italy) was done for fourteen eyes of five patients (II:5,III:10,III:12,III:25,IV:6) and two unaffected relatives (III:9,IV:9).

### Molecular genetic analysis

After obtaining written informed consent, peripheral blood samples were collected from eight patients, seven unaffected relatives and ten normal Chinese control subjects. Genomic DNA was extracted from blood lymphocytes using the ChargeSwitch® gDNA Blood Kits (Invitrogen, San Diego, CA). Exons 4 and 11 to 14 of the *TGFBI* gene were amplified and sequenced. Nucleotide Sequences were compared with the published *TGFBI* genomic sequence (GenBank NM_000358.1). The primers and PCR conditions used for amplification of TGFBI were listed in Table ﻿1.

**Table 1:** Lists of primers and PCR conditions used for amplification of *TGFBI*

Exon	Sequence of primers (5′ → 3′)	Product length (bp)	Annealing temperature (°C)
4	CCCCAGAGGCCATCCCTCCT (sense) CCGGGCAGACGGAGGTCATC (antisense)	225	58
11	GCCTTAATAACCCATCCCAGTGT (sense) TTTTCCCCATCCCAAGTCT (antisense)	427	62
12	CATTCCAGTGGCCTGGACTCTACTATC (sense) GGGGCCCTGAGGGATCACTACTT (antisense)	318	55
13	CCTCCTTGACCAGGCTAATTAC (sense) GGCTGCAACTTGAAGGTTGTG (antisense)	299	58
14	CTGTTCAGTAAACACTTGCT (sense) CTCTCCACCAACTGCCACAT (antisense)	361	60

﻿

### Surgical management

DLKPs using cryopreserved donor corneas were applied for four eyes of four patients (II:5,III:10,III:12,III:25). DLKP was performed using Yao’s hooking-and-detaching technique, which was developed by ourselves and described elsewhere [19, 20]. Briefly, after trephination using a trephine 7.25 to 8.25 mm in diameter, a small area of Descemet’s membrane (DM) exposure was created as a pocket at 12 o'clock around the trephined margin by hooking the stromal fibers using a unique forceps, through which viscoelastic material was injected between the posterior stromal layer and DM, and the full stroma layer was detached from DM extending to the full bed around the trephined margin. Stroma thereafter was removed in a single layer, making a complete exposure of DM in the full bed. If the primary pocket did not exactly reach the layer of DM, a second or third round of the hooking-and-detaching process was required until the total stroma was removed. A donor corneal button 0 to 0.5 mm oversize, obtained from a cryopreserved eye stored at −20 °C was sutured into the recipient bed by 10–0 nylon suture [20].

### Histologic, immunohistochemical and Ultrastructural study

Four lamellar dystrophic corneal buttons were obtained from DLKPs. The corneal buttons were bisected immediately after grafting. Three quarter of the button was fixed in 10 % neutral buffered formalin solution, dehydrated in ethanol and embedded in paraffin for general light microscopy and immunohistochemistry study. One quarter fixed with 2.5 % glutaraldehyde in phosphate-buffered saline (PBS), post-fixed in 1 % osmium tetroxide, dehydrated in ethanol, and embedded in epoxy resin for transmission electron microscopy. Two residue donor corneal buttons after penetrating keratoplasty (PKP) were used as normal control.

For light microscopy examination, paraffin sections were stained with hematoxylin and eosin, periodic acid-Schiff, Masson-Trichrome, and Congo red.

For immunohistochemistry study, consecutive 3 μm sections were deparaffinized and rehydrated following a standard procedure. Sections were incubated with a rabbit polyclonal anti-TGFBI antibody (Proteintech Group Inc., Chicago, IL), followed by incubation with Dako’s HRP, Rabbit/Mouse (ENV) reagent (Dako, Glestrop, Denmark) and visualized by Dako REAL™ DAB+ Chromogen.

For transmission electron microscopy, ultra-thin sections of 60 to 70 nm were cut with a diamond knife, mounted on copper grids, counterstained with uranyl acetate and lead citrate and examined by transmission electron microscope (JEM-1230, JEOL Company, Japan).

## Results

### Clinical characteristics

Eight members affected with corneal dystrophy were identified, and seven unaffected members of the four-generation pedigree were also examined. The proband (III:10), a 36-year-old woman visited our clinic on 2^nd^ Jun, 2011. She complained symptoms of recurrent eye red, pain and photophobia in both eyes starting in her early childhood. A progressive reduction in visual acuity occurred thereafter during her first decade when she was in primary school. She and her affected family relatives including her two sons had same symptoms of recurrent painful corneal erosion, which consistently occurred during the first decade of life.

**Fig. 2:**
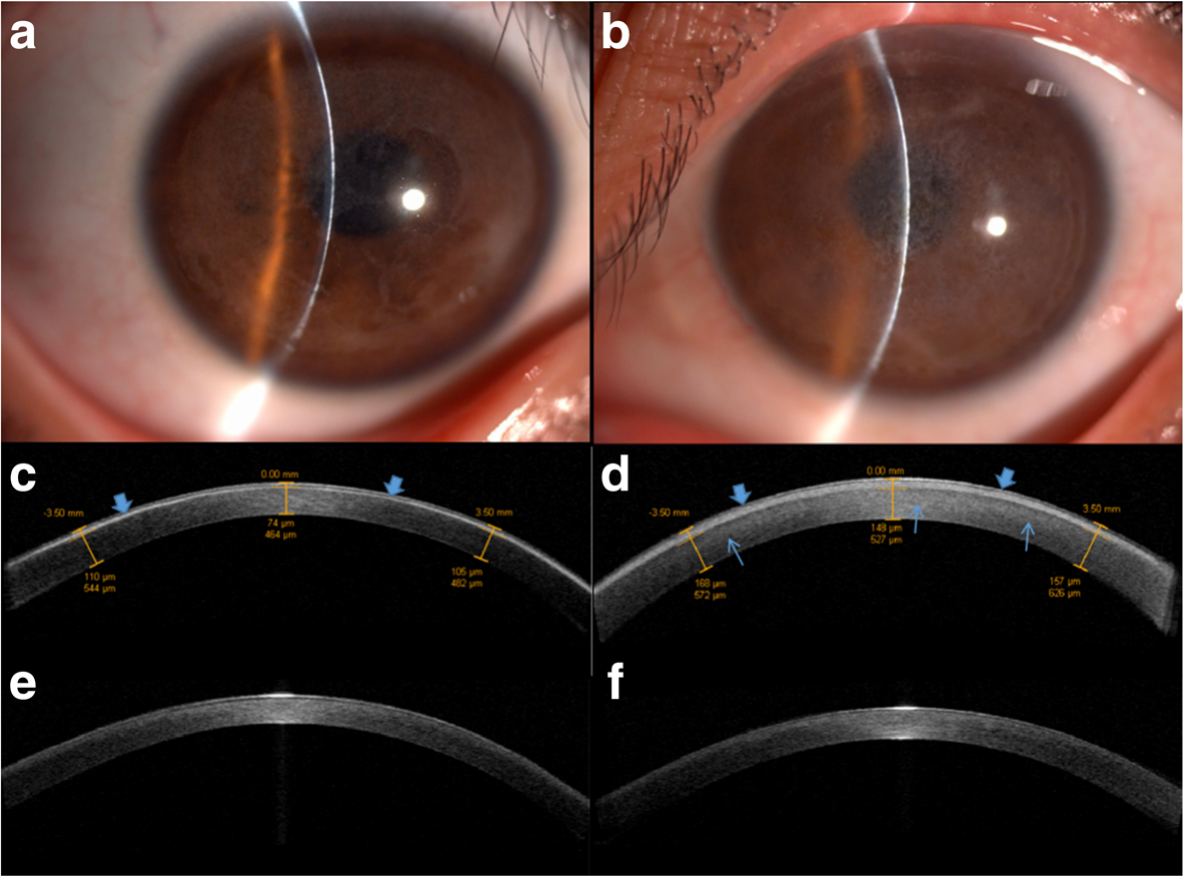
Slit lamp photographs and AS OCT images of the dystrophic corneas from IV:6 (**a**, **c**), III:10 (**b**, **d**) and age-matched healthy relatives IV:9 (**e**), and III:9 (**f**). **a** slit lamp photography of the right eye of IV:6. **b** slit lamp photography of the left eye of III:10. **c** AS OCT image of the same eye of (**a**). Arrowheads indicate hyper-reflective signals correspond with dystrophic opacity presenting at the level of Bowman’s layer. **d** AS OCT image of the same eye of (**b**). Hyper-reflective signals pointed by arrowheads involving both Bowman’s layer and superficial cornea. In underlying corneal stroma, the generally increased stromal reflectivity (pointed by blue arrows) is also observed. The thickness values of epithelium plus hyper-reflective signals and residue stroma thickness are labelled, respectively

Slit-lamp biomicroscopy revealed there are bilaterally, symmetric, age-dependent progressive corneal opacities in eight affected patients in the family. The opacities were diffuse gray-white sand like in confluent or non-confluent geographic morphology with a peripheral narrow strip of normal cornea. In two pediatric patients, the proband’s sons (IV:6, 6-year-old, and IV:7, 3-year-old) with visual acuity equal or more than 0.8, the corneal opacities in non-confluent geographic morphology were found (see Fig. 2a, IV:6). However, in all six adult patients involving II:2,II:5,II:9,III:10,III:12 and III:25 with visual acuity were less than 0.1, the opacity in confluent geographic morphology was observed (see Fig. 2b, III:10). Corneal epithelium usually was intact, but became irregular. No neovascularization and calcification, and no stromal lattice lines were observed.

### Anterior segment optical coherence tomography

AS OCT was performed on ten corneas of five affected subjects (II:5,II:10,III:12,III:25,IV:6). In pediatric patient (IV:6), we found a thin band of hyper-reflective signals correspond with dystrophic opacity presented at the level of Bowman’s layer (see Fig. 2c). The mean thickness value of the hyper-reflective signals is 42 ± 14um (five points in each cornea of both eyes were measured). However, the bands of hyper-reflective materials were much thicker in four examined adult patients (see Fig. 2d,III:10). The mean thickness value of the bands is 104 ± 35um (five points in each cornea of eight eyes in II:5,II:10,III:12,III:25 were measured). Compare to age-matched normal relatives (Fig. 2e,IV:9, and 2 F,III:9), generally increased stromal reflectivity in underlying corneal stroma in advanced eyes was also observed (Fig. 2d). The dystrophic corneas were thicker than normal, which were measured by AS OCT. The mean value of the whole central corneal thickness of is 545 ± 16um in pediatric patient and 608 ± 55um in adult patients, respectively.

### In vivo confocal microscopy

**Fig. 3:**
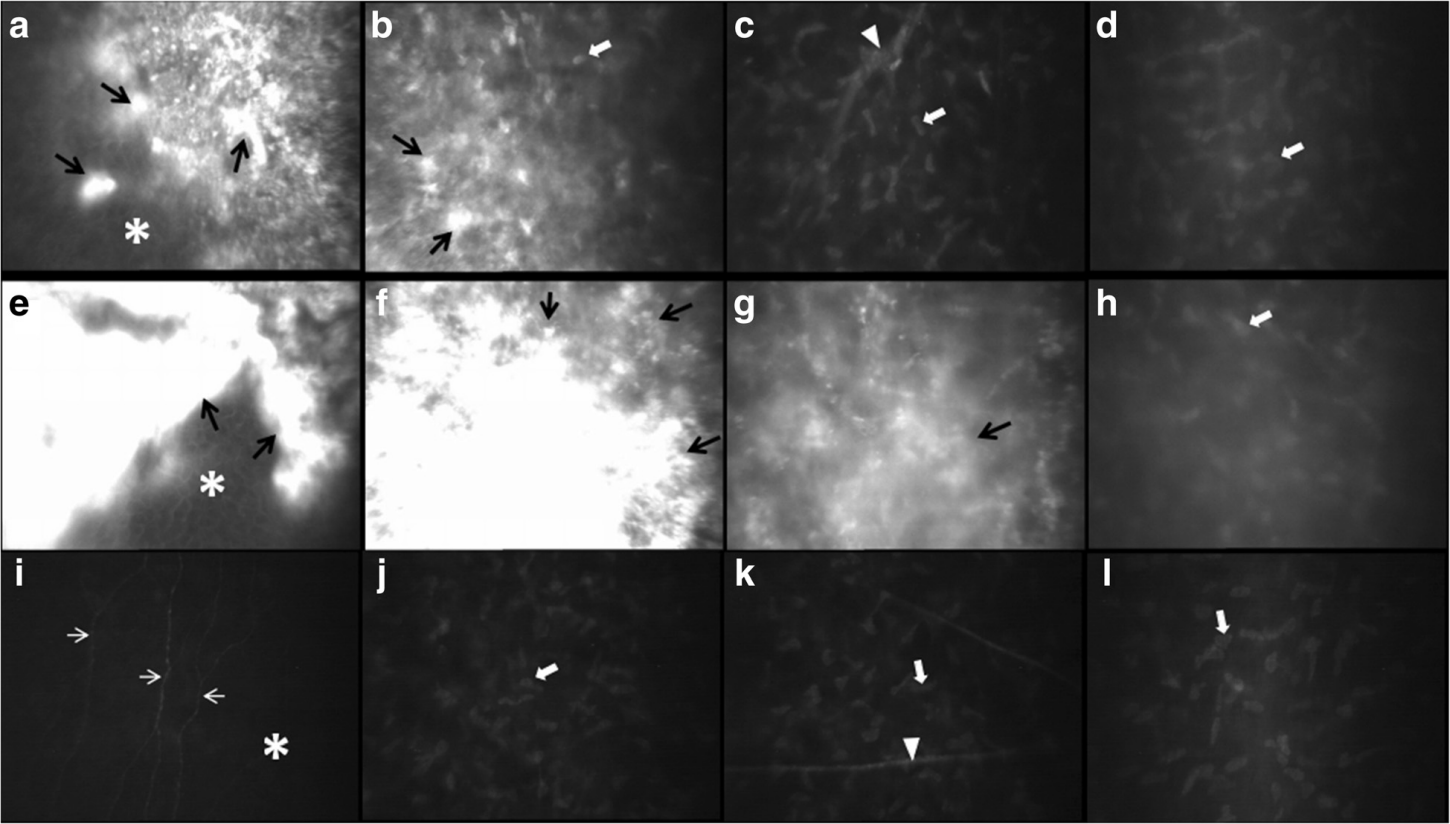
In vivo confocal microscopy photography of the dystrophic corneas from IV:6 (**a**, **b**, **c** and **d**),III:10 (**e**, **f**, **g** and **h**), and normal confocal images from healthy relative (III:9, **i**, **j**, **k** and **l**). Depths from the corneal surface are given. Panel (**a**, **e** and **i**) show appearances of epithelial basal cell layer and Bowman’s layer (depth 20-40 μm). Panel (**b**, **f** and **j**) demonstrate findings of anterior corneal stroma layer close to Bowman’s layer (depth 40-60 μm). Panel (**c**, **g** and **k**) display images of mid-stroma layer (depth 220 μm). Panel (**d**, **h** and **l**) show the pictures of deep stroma layer close to DM and endothelium (depth 500 μm). Black arrows point hyper-reflective granular aggregates. White asterisks indicate the epithelial basal cell layer. White arrows point sub-basal nerve plexus. Spindle-shaped material and stromal nerve pointed by *white triangle*, and keratocyte nuclei pointed by *white arrowheads*

By using in vivo confocal microscopy, in IV:6, we found homogeneous hyper-reflective granular aggregates diffused in the epithelial basal cell layer and Bowman’s layer of dystrophic cornea (Fig. 3a). Sub-basal nerve plexuses were not apparent (Fig. 3a). Some grayish aggregates presented in anterior stroma (Fig. 3b), while keratocyte nuclei and spindle-shaped materials in middle stroma were unremarkable (Fig. 3c). Keratocytes nuclei in posterior stroma (Fig. 3d), DM and the endothelium also appeared to be normal. In four adult patients, however, hyper-reflective deposits in affected eyes were inhomogeneous and varying sizes, and developed not only in superficial corneal layer (Fig. 3e and f,III:10), but also in mid-stroma (Fig. 3g,III:10). Sub-basal nerve plexuses and spindle-shaped materials were not observed. The deep posterior corneal stromal keratocyte nuclei and endothelium were also unremarkable (see Fig. 3h, III:10). Figure 3h, i, j and k are the normal confocal images from the healthy relative (III:9) corresponding to the epithelial basal cell and Bowman’s layer (Fig. 3h), and anterior, middle, posterior corneal stromal layer (Fig. 3i, j and k), respectively.

### Molecular analysis results

**Fig. 4:**
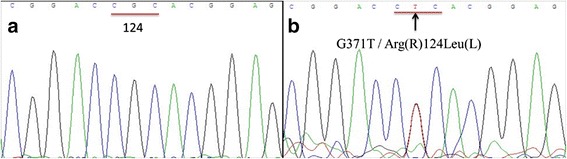
Direct sequencing of exon 4 of the *TGFBI* gene isolated from normal control and III:10. The sequence of a normal control (left) displayed CGC nucleotides in codon 124. The sequence of III:10 (right) showed a G to T transition at the second nucleotide position in the same codon (*arrow*), which results in an Arg124Leu substitution

Exon 4 and 11 to 14 of *TGFBI* gene from eight affected family members and their seven unaffected relatives were amplified and direct sequenced. The results showed that blood samples from eight affected family members consistently presented a single heterozygous G to T missense mutation at position 418 in exon 4 of *TGFBI* gene, leading to a change from Arginine (R) to Leucine (L) at codon 124 (Fig. 4). This Arg124Leu mutation co-segregated with RBCD within the family. None of the unaffected individuals and normal Chinese control subjects tested was positive for this mutation (Fig. 4). The mutation Arg555Gln in the fourth FAS1domain of TGFBI gene did not appear.

### Basis of diagnosis and surgical management

According to the typical clinical features and molecular analysis results, the diagnosis of RBCD was made for these patients with corneal dystrophy. The results of AS OCT and in vivo confocal microscopy showed that the dystrophic deposits involved not only in subepithelial and superficial stroma, but also in mid- or posterior stroma in advanced eyes with RBCD. On the basis of the findings, we did DLKP for four dystrophic eyes (II:5,III:10,III:12 and III:25).

### Light microscopy findings

**Fig. 5:**
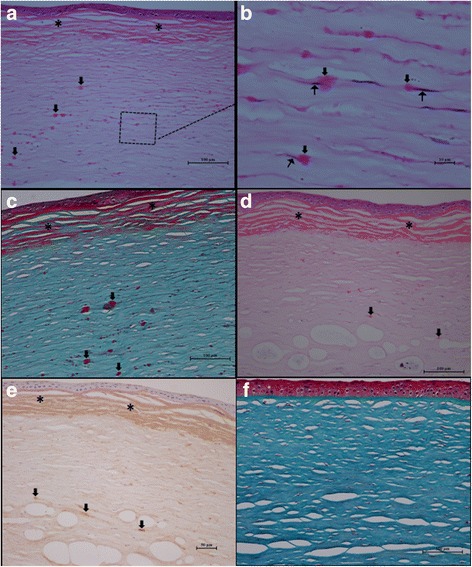
Histological sections of dystrophic corneal button from III:10 (**a**, **b** and **C**) and III:12 (**d** and **e**), and normal residue donor cornea (**f**). **a** Hematoxylin and Eosin staining (40X). **b** Higher magnification image (100X) of dystrophic aggregates presenting around the stromal keratocytes (*pointed by black arrows*). The region demarcated by the black rectangle in panel (**a**) is shown. **c** Masson-Trichrome staining (40X). **d** Hematoxylin and Eosin staining (40X). **e** Immunolabelling for TGFBI (40X). *Black asterisks* indicate dystrophic deposits stacking and producing three to five bands in superficial corneal layers. *Black arrowheads* point dystrophic aggregates scattering throughout the mid- to posterior stroma

In all four dystrophic corneal buttons obtained from DLKP surgeries, epithelium was markedly irregular in thickness and arrangement. Normal columnar basal cells and Bowman’s layer were absent, replaced by abnormal deposits (see Fig. 5a and c from III:10, and Fig. 5d from III:12). The junction between epithelium and underlying tissue loosed because of the deposits. As a result, epithelial cells were separated from the underlying tissue in some areas (Fig. 5a, c, d). The deposits might protrude into epithelium (Fig. 5c). Dystrophic deposits were characterized by staining bright red with Masson’s trichrome (Fig. 5c), and pink with hematoxylin and eosin (Fig. 5a, d) and Congo red, and were not stained by periodic acid-Schiff. Dystrophic deposits stacked and produced three to five bands parallel to the corneal collagen lamellae in superficial corneal layers. Meanwhile, the number of stromal keratocytes decreased significantly in superficial stroma. In underlying stroma, however, the dystrophic aggregates were granular or dot- like and heavily scattered throughout the mid- to posterior stroma (see Fig. 5a, c, d). Only one- third or one quarter of the deep posterior corneal stroma remained normal in all four dystrophic corneal buttons. The most interesting finding was some mutated proteins presenting around the stromal keratocytes (see Fig. 5b, III:10), which had never been reported before in the literature. Immunohistochemistry study showed that the dystrophic deposits were strongly positive stained with anti-TGFBI antibody (see Fig. 5e,III:12). Figure 5f showed the image of normal control from the residue donor cornea which was stained with Masson’s trichrome.

### Transmission electron microscopy findings

**Fig. 6:**
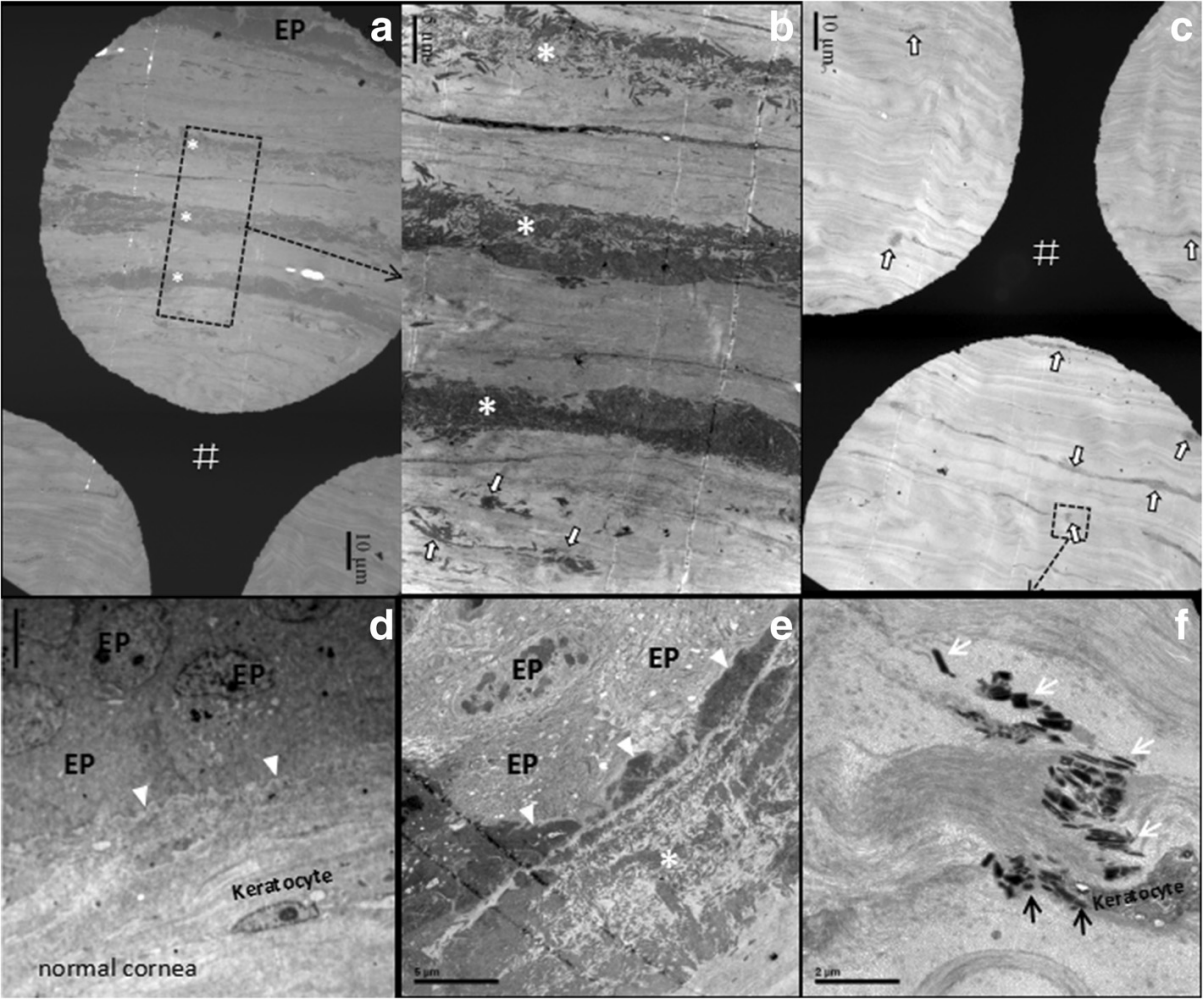
Transmission electron microscopy pictures of dystrophic corneal buttons from III:10 (**a**, **b**, **c**, **e** and **f**) and normal residue donor cornea (**d**). **a** low magnification image (1200X) overview of epithelium and anterior corneal stroma. White hash marks denote the mounted copper grids. **b** Higher magnification image (3000X) view of a characteristic pattern of three bands including numerous electron-dense, rod-shaped bodies parallel to the epithelial layer in superficial cornea (*pointed by white asterisks*). The region demarcated by the black rectangle in panel (**a**) is shown. In underlying stroma, clusters of rod-shaped bodies are aggregate (*pointed by arrowheads*). **c** Low magnification (1200X) view of clusters of deposits scattered in posterior corneal stroma (pointed by arrowheads). **d** TEM image (3650X) of normal epithelial basal cells (indicated by EP), basement membrane (pointed by triangle) and keratocyte. **e** Higher magnification (6000X) view of corneal epithelium (indicated by EP) and superficial dystrophic deposits. The deposits protrude into extracellular of epithelium (*pointed by triangle*), other than intracellular of epithelium. **f** High magnification (15000X) of rod-shaped bodies presenting extracellular (*pointed by white arrows*) and intracellular (*pointed by black arrows*) of stromal keratocytes. The region demarcated by the black rectangle in panel (**c**) is shown

Electron microscopy revealed multiple irregular, electron-dense, 100 to 500 nm wide rod-shaped bodies in the dystrophic corneas (see Fig. 6b and f, III:10). Structure and appearance of the rod-shaped bodies were similar to those described in the classic form of granular corneal dystrophy (GCD). Compare to normal control from the residue donor cornea (see Fig. 6d), the epithelial basement membrane and Bowman’s layer in the dystrophic corneas were absent (see Fig. 6e,III:10). The deposits might protrude into extracellular of epithelium (Fig. 6e). In subepithelial and superficial cornea, the deposits aggregated and produced a characteristic pattern of three to five bands parallel to the epithelial layer (see Fig. 6a, b). In underlying corneal stroma, however, clusters of rod-shaped bodies were scattered between the stromal lamellae and usually around the stromal keratocytes which were similar to those seen under light microscopy (see Fig. 6b, c and f, III:10). The most important finding was a phenomenon of some rod-shaped bodies intracellular or extracellular of the stromal keratocyte suggesting the close relationship between mutated proteins and stromal keratocyte (Fig. 6f). Meanwhile, the stromal collagen fibrils were abnormally ruptured or directions of the collagen fibrils were changed because of the deposits aggregation (Fig. 6f). The last cluster of rod-shaped structures was deeply located in the posterior corneal stroma. The distance from the surface of epithelium to it was about 300–400 μm, based on the length of mounted copper grids. No curly fibers were observed in all four tested dystrophic corneal buttons.

## Discussion

Two forms of Bowman’s layer corneal dystrophies (CDB) have been recognized: Reis–Bücklers (CDB type I) and Thiel-Behnke corneal dystrophies (CDB type II, OMIM 602082). Criteria have been available to segregate RBCD from TBCD as following. Clinically, Reis–Bücklers corneal dystrophy is characterized by confluent geographic opacities whereas Thiel-Behnke corneal dystrophy by honeycomb-shaped opacities. Histopathologically, RBCD is identified by the presence of “rod -shaped bodies” and TBCD by the presence of “curly fibers”. Genetically, RBCD is caused by an R124L kerato-epithelin (KE) mutation while TBCD by an Arg555Gln KE mutation. Briefly, RBCD with geographic opacities had an R124L mutation, while TBCD with honeycomb-shaped opacities had an Arg555Gln mutation. Therefore, the diagnosis of these patients with autosomal dominant inherited corneal dystrophy in the present study was RBCD without any doubt.

RBCD, also known as granular corneal dystrophy (GCD) type III or superficial variant of GCD because the mutated protein in RBCD and GCD possess the same rod-shaped configuration. Up to now, the source of mutated KE deposits in GCD and other TGFBIp-related corneal dystrophies such as lattice dystrophy is still controversial. Initially, it was believed that the abnormal deposits in stroma derived from stromal keratocytes [21]. Recently, concerning the genetic and molecular aspects of KE, more reports supported the stromal dystrophic deposits were epithelial in origin [9–12]. KE was predominantly found on corneal epithelial cells [7], and it was an adhesion protein of 653 amino acid whose mass was similar to that of albumin. Such size of protein would permit the diffusion across the stroma was assumed. However, a clinical study seems to refute the hypothesis of epithelial genesis because limbo-keratoplasty with replacement of recipient epithelium by donor epithelium had no more effect in preventing recurrence of granular and lattice corneal dystrophies than PKP [11]. In addition, in 2006, we reported clinical and histological findings in a recurrent case of lattice dystrophy after primary DLKP [22]. We found recurrent amyloidosis was mainly located in residual recipient stroma just before Descemet membrane and not in the donor graft. The epithelial genesis hypothesis cannot explain that the epithelium-derived KE diffuse across the full thickness of the donor graft without any trace to deposit the residual stromal layer. Thus, we speculated recurrent amyloidosis of lattice corneal dystrophy were stromal genesis. In the present study, we found some rod-shaped bodies were intracellular of the stromal keratocyte (Fig. 6f). The deposits might protrude into extracellular of epithelium, but were not in the cytoplasm of epithelial cells (Fig. 6e). Therefore, the study may offer evidence demonstrating the mutated TGFBIp in RBCD corneas are of keratocyte origin. More designed studies would be requied to confirm the findings in different families or ethnicities in the future.

With regard to choosing a corneal surgery for recovery of corneal clarity and visual rehabilitation when the opacity of dystrophic cornea is more dense and severe. Superficial keratectomy or PTK is a highly recommended treatment option in literature [15–17], because it is widely accepted that the pathologic changes only involve the superficial cornea in RBCD eyes. However, there is almost always a recurrence of RBCD after PTK within several months post-operatively [18]. Recurrences of other TGFBIp-related corneal dystrophy such as Thiel-Behnke, granular and lattice dystrophies also have been reported after PTK in papers [18, 23]. Compare to TBCD, the recurrence of RBCD occurs earlier and with a more severe degree of disease after PTK [24]. The new histopathologic and ultrastructural findings in the present study may certainly aid in understanding why RBCD has a higher rate of recurrence after PTK. In all four dystrophic corneal buttons, we found the stacked high-density deposits definitely located in superficial cornea, which could be removed by PTK. In mid- or posterior corneal stroma, however, the diffused mutated protein deposits also were observed. Those diffused deposits certainly were left in the residual corneal stroma if the patients were treated by PTK. Besides, the hypothesis that laser-induced wound healing responses may accelerate TGFBIp deposition have been verified in papers [23, 25]. In the present study, we applied DLKP using cryopreserved donor corneas to treat four eyes of four patients, and no recurrence was observed during a 4-year period of follow-up. We analyzed that DLKP using cryopreserved donor corneas had three merits to delay the recurrence of RBCD. Firstly, the central dystrophic recipient corneal stroma is completely removed by DLKP. Secondly, there is no live keratocyte in the graft stroma of cryopreserved tissue, and keratocyte repopulation was very slow after DLKP. At last, there is minimal inflammation reaction without allogenic rejection in the eye treated by DLKP using cryopreserved donor corneas.

## Conclusions

The present study demonstrates new histopathologic and ultrastructural findings in RBCD as following. Firstly, in advanced RBCD, we found the mutated protein not only in the superficial corneal layer, but also in mid- and posterior-stroma using AS-OCT, in vivo confocal microscopy, light and transmission electron microscopy. DLKP could be a viable treatment option for advanced RBCD to avoid recurrence. Secondly, the mutated proteins were found both extracellular and intracellular of the stromal keratocyte by transmission electron microscopy. The finding gains evidence demonstrating the mutated TGFBIp in RBCD corneas are of keratocyte origin.

## Abbreviations

AS OCT: Anterior segment optical coherence tomography
DLKP: Deep anterior lamellar keratoplasty
PKP: Penetrating keratoplasty
PTK: Excimer lasers phototherapeutic keratectomy
RBCD: Reis-Bücklers corneal dystrophy
TBCD: Thiel-Behnke corneal dystrophy
*TGFBI*: Transforming growth factor induced gene

## References

[1] Rice NS, Ashton N, Jay B, Blach RK (1968). Reis-Bücklers’ dystrophy. A clinico- pathological study. Br J Ophthalmol.

[2] Küchle M, Green WR, Völcker HE, Barraquer J (1995). Reevaluation of corneal dystrophies of Bowman’s layer and the anterior stroma (Reis-Bücklers and Thiel-Behnke types): a light and electron microscopic study of eight corneas and a review of the literature. Cornea.

[3] Okada M, Yamamoto S, Tsujikawa M, Watanabe H, Inoue Y, Maeda N, Shimomura Y, Nishida K, Quantock AJ, Kinoshita S, Tano Y (1998). Two distinct kerato-epithelin mutations in Reis-Bücklers corneal dystrophy. Am J Ophthalmol.

[4] Streeten BW, Qi Y, Klintworth GK, Eagle RC, Strauss JA, Bennett K (1999). Immunolocalization of beta ig-h3 protein in 5q31-linked corneal dystrophies and normal corneas. Arch Ophthalmol.

[5] Dighiero P, Valleix S, D’Hermies F, Drunat S, Ellies P, Savoldelli M, Pouliquen Y, Delpech M, Legeais JM, Renard G (2000). Clinical, histologic, and ultrastructural features of the corneal dystrophy caused by the R124L mutation of the BIGH3 gene. Ophthalmology.

[6] Skonier J, Neubauer M, Madisen L, Bennett K, Plowman GD, Purchio AF (1992). cDNA cloning and sequence analysis of βig-h3, a novel gene induced in a human adenocarcinoma cell line after treatment with transforming growth factor-β. DNA Cell Biol.

[7] Escribano J, Hernando N, Ghosh S, Crabb J, Coca-Prados M (1994). cDNA from human ocular ciliary epithelium homologous to beta ig-h3 is preferentially expressed as an extracellular protein in the corneal epithelium. J Cell Physiol.

[8] Rawe IM, Zhan Q, Burrows R, Bennett K, Cintron C (1997). Beta-ig.Molecular cloning and in situ hybridization in corneal tissues. Invest Ophthalmol Vis Sci.

[9] Akhtar S, Meek KM, Ridgway AE, Bonshek RE, Bron AJ (1999). Deposits and proteoglycan changes in primary and recurrent granular dystrophy of the cornea. Arch Ophthalmol.

[10] Morand S, Buchillier V, Maurer F, Bonny C, Arsenijevic Y, Munier FL, Schorderet DF (2003). Induction of apoptosis in human corneal and HeLa cells by mutated BIGH3. Invest Ophthalmol Vis Sci.

[11] Spelsberg H, Reinhard T, Henke L, Berschick P, Sundmacher R (2004). Penetrating limbo-keratoplasty for granular and lattice corneal dystrophy: survival of donor limbal stem cells and intermediate-term clinical results. Ophthalmology.

[12] Yellore VS, Rayner SA, Aldave AJ (2011). TGFB1-induced extracellular expression of TGFBIp and inhibition of TGFBIp expression by RNA interference in a human corneal epithelial cell line. Invest Ophthalmol Vis Sci.

[13] Choi SI, Yoo YM, Kim BY, Kim TI, Cho HJ, Ahn SY, Lee HK, Cho HS, Kim EK (2010). Involvement of TGF-{beta} receptor- and integrin-mediated signaling pathways in the pathogenesis of granular corneal dystrophy II. Invest Ophthalmol Vis Sci.

[14] Kim TI, Kim H, Lee DJ, Choi SI, Kang SW, Kim EK (2011). Altered mitochondrial function in type 2 granular corneal dystrophy. Am J Pathol.

[15] Klintworth GK (2009). Corneal dystrophies. Orphanet J Rare Dis.

[16] Nassaralla BA, Garbus J, McDonnell PJ (1996). Phototherapeutic keratectomy for granular and lattice corneal dystrophies at 1.5 to 4 years. J Refract Surg.

[17] Reddy JC, Rapuano CJ, Nagra PK, Hammersmith KM (2013). Excimer laser phototherapeutic keratectomy in eyes with corneal stromal dystrophies with and without a corneal graft. Am J Ophthalmol.

[18] Dinh R, Rapuano CJ, Cohen EJ, Laibson PR (1999). Recurrence of corneal dystrophy after excimer laser phototherapeutic keratectomy. Ophthalmology.

[19] Yao YF (2008). A novel technique for performing full-bed deep lamellar keratoplasty. Cornea.

[20] Wu SQ, Zhou P, Zhang B, Qiu WY, Yao YF (2012). Long-term comparison of full-bed deep lamellar keratoplasty with penetrating keratoplasty in treating corneal leucoma caused by herpes simplex keratitis. Am J Ophthalmol.

[21] Wittebol-Post D, van der Want JJ, van Bijsterveld OP (1987). Granular dystrophy of the cornea (Groenouw’s type I). Is the keratocyte the primary source after all?. Ophthalmologica.

[22] Yao YF, Jin YQ, Zhang B, Zhou P, Zhang YM, Qiu WY, Mou SL, Wu LQ (2006). Recurrence of lattice corneal dystrophy caused by incomplete removal of stroma after deep lamellar keratoplasty. Cornea.

[23] Dogru M, Katakami C, Nishida T, Yamanaka A (2001). Alteration of the ocular surface with recurrence of granular/avellino corneal dystrophy after phototherapeutic keratectomy: report of five cases and literature review. Ophthalmology.

[24] Hieda O, Kawasaki S, Wakimasu K, Yamasaki K, Inatomi T, Kinoshita S (2013). Clinical outcomes of phototherapeutic keratectomy in eyes with Thiel-Behnke corneal dystrophy. Am J Ophthalmol.

[25] Aldave AJ, Sonmez B, Forstot SL, Rayner SA, Yellore VS, Glasgow BJ (2007). A clinical and histopathologic examination of accelerated TGFBIp deposition after LASIK in combined granular-lattice corneal dystrophy. Am J Ophthalmol.

